# Radiomics nomogram based on optimal VOI of multi-sequence MRI for predicting microvascular invasion in intrahepatic cholangiocarcinoma

**DOI:** 10.1007/s11547-023-01704-8

**Published:** 2023-09-07

**Authors:** Xijuan Ma, Xianling Qian, Qing Wang, Yunfei Zhang, Ruilong Zong, Jia Zhang, Baoxin Qian, Chun Yang, Xin Lu, Yibing Shi

**Affiliations:** 1 Department of Radiology, Xuzhou Central Hospital, Xuzhou Clinical School of Xuzhou Medical University, No. 199 Jiefang South Road, Quanshan District, Xuzhou, 221009 Jiangsu People’s Republic of China; 2 Department of Radiology, Zhongshan Hospital, Fudan University, No. 180 Fenglin Rd, Shanghai, 200032 People’s Republic of China; 3 Shanghai Institute of Medical Imaging, No. 180 Fenglin Rd, Shanghai, 200032 People’s Republic of China; 4 Department of Cancer Center, Zhongshan Hospital, Fudan University, No. 180 Fenglin Rd, Shanghai, 200032 People’s Republic of China; 5 Graduate Department, Bengbu Medical College, Bengbu, 233000 Anhui People’s Republic of China; 6 Central Research Institute, United Imaging Healthcare, No. 2258 Chengbei Rd, Shanghai, 201807 People’s Republic of China; 7 Huiying Medical Technology, Huiying Medical Technology Co., Ltd, Room A206, B2, Dongsheng Science and Technology Park, Haidian District, Beijing City, 100192 People’s Republic of China; 8 Department of Radiology, Shanghai Geriatric Medical Center, No. 2560 Chunshen Rd, Shanghai, 201104 People’s Republic of China

**Keywords:** Intrahepatic cholangiocarcinoma, Microvascular invasion, Magnetic resonance imaging, Nomogram

## Abstract

**Objective:**

Microvascular invasion (MVI) is a significant adverse prognostic indicator of intrahepatic cholangiocarcinoma (ICC) and affects the selection of individualized treatment regimens. This study sought to establish a radiomics nomogram based on the optimal VOI of multi-sequence MRI for predicting MVI in ICC tumors.

**Methods:**

160 single ICC lesions with MRI scanning confirmed by postoperative pathology were randomly separated into training and validation cohorts (TC and VC). Multivariate analysis identified independent clinical and imaging MVI predictors. Radiomics features were obtained from images of 6 MRI sequences at 4 different VOIs. The least absolute shrinkage and selection operator algorithm was performed to enable the derivation of robust and effective radiomics features. Then, the best three sequences and the optimal VOI were obtained through comparison. The MVI prediction nomogram combined the independent predictors and optimal radiomics features, and its performance was evaluated via the receiver operating characteristics, calibration, and decision curves.

**Results:**

Tumor size and intrahepatic ductal dilatation are independent MVI predictors. Radiomics features extracted from the best three sequences (T1WI-D, T1WI, DWI) with VOI_10mm_ (including tumor and 10 mm peritumoral region) showed the best predictive performance, with AUC_TC_ = 0.987 and AUC_VC_ = 0.859. The MVI prediction nomogram obtained excellent prediction efficacy in both TC (AUC = 0.995, 95%CI 0.987–1.000) and VC (AUC = 0.867, 95%CI 0.798–0.921) and its clinical significance was further confirmed by the decision curves.

**Conclusion:**

A nomogram combining tumor size, intrahepatic ductal dilatation, and the radiomics model of MRI multi-sequence fusion at VOI_10mm_ may be a predictor of preoperative MVI status in ICC patients.

**Supplementary Information:**

The online version contains supplementary material available at 10.1007/s11547-023-01704-8.

## Introduction

Intrahepatic cholangiocarcinoma (ICC) is the second leading primary liver cancer with increasing worldwide incidence in recent years [1–4]. ICC classification is based on its macroscopic growth pattern and can be divided into mass-forming (MF), periductal invading, intraductal developing, and mixed-type. Among these, the MF-ICC is the most prevalent and accounts for 60% [5, 6]. ICC patient prognosis is relatively poor, and surgery remains the most effective intervention [7–9].

MVI is characterized by cancer cell nests within vessels of surrounding liver parenchyma [10–12], and it is an independent indicator of overall survival (OS) of ICC patients, particularly, early recurrence and poor prognosis [13, 14]. Tang et al*.* [15] suggested that MVI is inversely related to OS and disease-free survival of ICC patients. Moreover, MVI can influence individualized treatment options, for instance, ICC patients without MVI do not require adjuvant chemotherapy following R0 resection [11]. Currently, MVI is only identified via postoperative pathology [11, 14, 16]. Although certain laboratory blood evaluations and radiological characteristics (e.g., ADC value and tumor size) can predict MVI status in ICC, the optimal preoperative imaging criteria for MVI detection remains inconclusive owing to controversial results [15–17].

Radiomics has been a research hotspot in recent years, which can enhance diagnostic proficiency via high-throughput medical imaging profile selection [18], and can also be applied to preoperative MVI prediction. In terms of the MVI-based prediction of ICC, Zhou et al*.* [19] reported the potential of seven wavelet profiles obtained from presurgical MRI images, with an area under the curve (AUC) of 0.873. Qian et al. [20] established a prediction nomogram that incorporates two imaging features and the final radiomics model and achieves excellent MVI prediction with an AUC of 0.953. Pathologically, MVI occurs in peritumoral regions, whereas, in past studies, radiomics features were only retrieved from inside the ICC tumor. To predict MVI or treatment response, some studies extracted the radiomics features from the surrounding liver tissue of hepatocellular carcinoma (HCC) [21–23]. Chong et al. [21] suggested that radiomics models incorporating both intratumoral and 10 mm peritumoral regions can effectively predict the MVI status of HCC. Currently, there is no literature to systematically evaluate the radiomics features of the multiple peritumoral ranges of ICC. Therefore, we attempt to investigate the impact of different regions on MVI predictive outcome by radially expanding the distances at 8, 10, and 12 mm from the tumor boundary. In this study, we delineated different volumes of interest (VOIs) from four different regions of intratumoral and peritumoral tissue on six MR sequences and extracted radiomics features. Then, the optimal VOI and sequence were determined through comparison to select the best combination for constructing the radiomics model for predicting MVI. In summary, our research aimed to develop a radiomics nomogram for the prediction of MVI status in MF-ICC, which integrates the radiomics model and the independent MVI predictors of clinical and MR imaging characteristics.

## Materials and methods

This retrospective diagnostic study received ethical approval from two institutions. And written informed consent requirement was waived.

### Patients

**Fig. 1:**
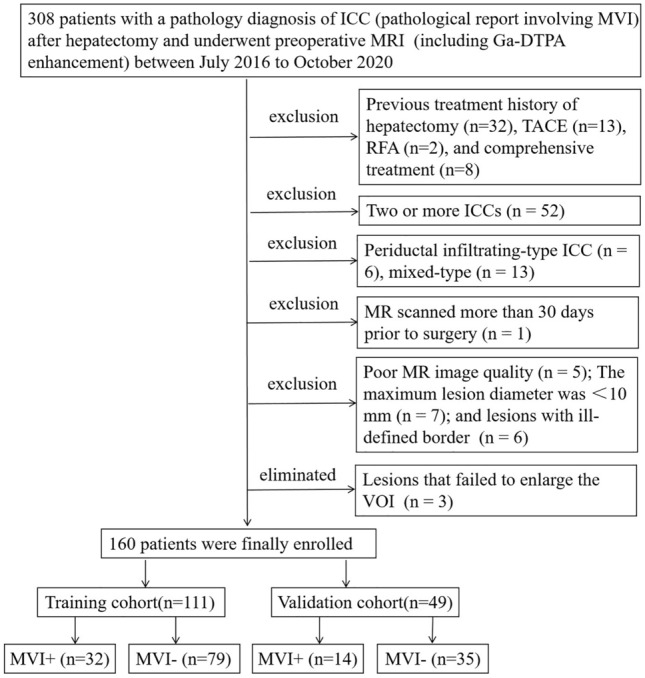
Study flowchart of the enrolled patients

Between July 2016 to October 2020, 160 patients with postoperatively and pathologically confirmed ICC and preoperative enhanced MRI scanning were enrolled in this study. The inclusion criteria (Fig. 1) were as follows: (1) no history of liver cancer therapy; (2) single lesion; (3) pathology confirmed MF-ICC; (4) MRI scanning was performed within 30 days before surgery; (5) satisfactory imaging quality without obvious artifacts; (6) the largest lesion diameter was ≥ 10 mm; (7) the lesions can be identified in MRI images, and can be expanded the peritumoral range successfully. Three lesions cannot be expanded by software, because the spatial resolution of some sequence images was lower than the external expansion value. According to the ratio in the previous studies focused on radiomics and primary liver cancer [21, 22], all enrolled patients were randomly separated into training cohort (TC, *n* = 111) and validation cohort (VC, *n* = 49) at a ratio of 7:3.

### Clinicopathological characteristics

Presurgical laboratory indexes and demographic data (Table 1) were obtained from the medical documentation system, including age, sex, history of hepatitis B virus (HBV), alpha-fetoprotein (AFP), carcinoembryonic antigen (CEA), and carbohydrate antigen 19-9 (CA199). All surgical tumor specimens were obtained by hepatectomy and sampled via a 7-point baseline sampling protocol [12]. The recorded pathological characteristics included the number of lesions, Edmondson-Steiner grade, and MVI status. MVI was defined as the evidence of a tumor cell nest (over 50 suspended tumor cells) in the portal and hepatic veins, or large capsular vessels in the peritumoral regions on microscopy [12, 24]. Tumor histological grades included well (G1), moderately (G2), and poorly (G3) differentiated [25]. Pathological characteristics were evaluated unanimously by two independent pathologists in each hospital, and a consensus would be reached if there were any disagreements. Quantitative data were obtained by averaging the results from the two pathologists independently reading.

**Table 1:** Comparison of MVI status and clinical and imaging characteristics in patients of training and validation cohorts

Characteristics	Training cohort (*n* = 111)			Validation cohort (*n* = 49)			*p*-Inter
	MVI (−), (*n* = 79)	MVI (+), (*n* = 32)	*p*-Intra	MVI (−), (*n* = 35)	MVI (+), (*n* = 14)	*p*-Intra	
**Clinical features**							
Age, years*	59.77 (12.05)	59.94 (11.37)	0.947	60.97 (11.09)	62.29 (9.42)	0.698	0.438
Gender			1			0.878	1.000
Female	21 (26.6)	9 (28.1)		10 (28.6)	3 (21.4)		
Male	58 (73.4)	23 (71.9)		25 (71.4)	11 (78.6)		
HBV			0.919			0.211	0.033
Negative	47 (59.5)	18 (56.2)		16 (45.7)	3 (21.4)		
Positive	32 (40.5)	14 (43.8)		19 (54.3)	11 (78.6)		
AFP			1			0.787	0.091
< 20 ng/ml	70 (88.6)	29 (90.6)		28 (80.0)	10 (71.4)		
≥ 20 ng/ml	9 (11.4)	3 (9.4)		7 (20.0)	4 (28.6)		
CEA			0.250			0.263	0.105
< 5 ng/ml	64 (81.0)	22 (68.8)		33 (94.3)	11 (78.6)		
≥ 5 ng/ml	15 (19.0)	10 (31.2)		2 (5.7)	3 (21.4)		
CA199			0.194			0.179	0.572
< 34U/ml	47 (59.5)	14 (43.8)		24 (68.6)	6 (42.9)		
≥ 34U/ml	32 (40.5)	18 (56.2)		11 (31.4)	8 (57.1)		
Edmondson-Steiner grade			0.051			0.117	0.746
II	29 (36.7)	5 (15.6)		15 (42.9)	2 (14.3)		
III	50 (63.3)	27 (84.4)		20 (57.1)	12 (85.7)		
**MR imaging features**							
Tumor size, mm*	40.28 (21.95)	59.49 (27.77)	**< 0.001**	43.80 (21.15)	52.40 (21.78)	0.208	0.915
Tumor morphology			0.048			0.203	0.855
(Hemi-)spherical and oval	38 (48.1)	8 (25.0)		13 (37.1)	5 (35.7)		
Lobulated	26 (32.9)	18 (56.2)		17 (48.6)	4 (28.6)		
Irregular	15 (19.0)	6 (18.8)		5 (14.3)	5 (35.7)		
SI on T1WI			0.227			1	0.797
Low	78 (98.7)	30 (93.8)		34 (97.1)	14 (100.0)		
Moderate	1 (1.3)	1 (3.1)		1 (2.9)	0 (0.0)		
High	0 (0.0)	1 (3.1)		0 (0.0)	0 (0.0)		
SI on T2WI-FS			1			0.659	0.282
Low	0 (0.0)	0 (0.0)		1 (2.9)	0 (0.0)		
Moderate	3 (3.8)	1 (3.1)		1 (2.9)	0 (0.0)		
High	76 (96.2)	31 (96.9)		33 (94.3)	14 (100.0)		
Target sign on T2WI-FS			0.555			0.189	0.041
Negative	58 (73.4)	21 (65.6)		16 (45.7)	10 (71.4)		
Positive	21 (26.6)	11 (34.4)		19 (54.3)	4 (28.6)		
Target sign on DWI			0.281			**0.007**	0.125
Negative	50 (63.3)	16 (50.0)		11 (31.4)	11 (78.6)		
Positive	29 (36.7)	16 (50.0)		24 (68.6)	3 (21.4)		
Rim enhancement on T1WI-A			1			0.651	0.324
Negative	18 (22.8)	7 (21.9)		4 (11.4)	3 (21.4)		
Positive	61 (77.2)	25 (78.1)		31 (88.6)	11 (78.6)		
Enhancement pattern			0.540			0.179	0.524
Gradual and filling	57 (72.2)	26 (81.2)		26 (74.3)	10 (71.4)		
Persistent enhancement	10 (12.7)	2 (6.2)		7 (20.0)	1 (7.1)		
Wash-in and wash-out	12 (15.2)	4 (12.5)		2 (5.7)	3 (21.4)		
LI-RADS			0.287			0.063	0.642
LR-3	1 (1.3)	0 (0.0)		0 (0.0)	0 (0.0)		
LR-4	4 (5.1)	0 (0.0)		1 (2.9)	0 (0.0)		
LR-5	8 (10.1)	2 (6.2)		0 (0.0)	2 (14.3)		
LR-M	66 (83.5)	29 (90.6)		34 (97.1)	12 (85.7)		
LR-TIV	0 (0.0)	1 (3.1)		0 (0.0)	0 (0.0)		
Intrahepatic duct dilatation			**0.002**			0.487	1.000
Negative	56 (70.9)	12 (37.5)		23 (65.7)	7 (50.0)		
Positive	23 (29.1)	20 (62.5)		12 (34.3)	7 (50.0)		
Hepatic capsular retraction			0.515			1	0.920
Negative	49 (62.0)	17 (53.1)		20 (57.1)	8 (57.1)		
Positive	30 (38.0)	15 (46.9)		15 (42.9)	6 (42.9)		
Visible vessel penetration			0.700			1	0.011
Negative	37 (46.8)	13 (40.6)		8 (22.9)	3 (21.4)		
Positive	42 (53.2)	19 (59.4)		27 (77.1)	11 (78.6)		
Peripherally hepatic enhancement			0.316			0.613	0.827
Negative	32 (40.5)	17 (53.1)		13 (37.1)	7 (50.0)		
Positive	47 (59.5)	15 (46.9)		22 (62.9)	7 (50.0)		

Data are shown as the number of patients and percentage in parentheses unless otherwise stated

*Data are means and standard deviations in parentheses

The bold values are statistically significant with *p* < 0.05

### MR imaging

All patients were examined with enhanced MRI scanning. Gadopentetate dimeglumine (Haipu Pharmaceutical Co., Ltd.) was administered at 0.1 mmol/kg followed by a 20 ml saline flush at 2 ml/s using a power injector. Patients were scanned using the following nine MRI scanners: a 1.5T UIHMR560 scanner (United Imaging Healthcare, Shanghai, China) for 62 patients; a 3.0T UIHMR770 scanner (United Imaging Healthcare, Shanghai, China) for 38 patients; a Magnetom Avanto 1.5T imager (Siemens Healthcare, Erlangen, Germany) for 29 patients; a Magnetom Aera 1.5T imager (Siemens Healthcare, Erlangen, Germany) for 16 patients; a Magnetom Verio 3.0T MRI System (Siemens Healthcare, Erlangen, Germany) for 3 patients; an Achieva 1.5T MRI System (Philips Medical Systems, Best, Netherlands) for 3 patients; and three 3.0T Discovery750 MRI Systems (GE Healthcare, Milwaukee, WI, USA) for 9 patients. The typical scanning sequences used in our study were completed as follows: axial T2-weighted breath-hold fat-suppressed fast spin echo sequence (T2WI-FS), and diffusion-weighted imaging (DWI) conducted via free-breath single-shot spin-echo-planar sequence (*b* = 0, 800 mm^2^/s). Breath-hold fat-suppressed 3D T1-weighted quick spoiled gradient echo sequences (T1WI-FS), including mask phase (T1WI), axial arterial phase (T1WI-A), axial portal venous phase (T1WI-V), and axial delayed phase (T1WI-D), were taken at the 20–30 s [26], 60–90 s, and 160–180 s, respectively, following contrast agent administration. The parameters are shown in detail in Supplemental Table S1.

### Qualitative and quantitative MRI analyses

The following imaging characteristics of the tumor were assessed: (a) Tumor size, described as the largest axial diameter on T1WI-D (T1WI-V was selected when the edge of the T1WI-D was not clear and could not be measured); (b) Tumor morphology (spherical/hemispherical/oval, lobulated, and irregular); (c) Signal intensity (SI) of tumor (relative to the surrounding liver) on T1WI-FS, T2WI-FS and DWI, such as, hypo-, iso-, and hyperintensity; (d) Target sign on T2WI-FS and DWI, representing peripheral hyperintense with central isointense/hypointense [27]; I Intrahepatic duct dilatation; (f) Capsular retraction; (g) Rim enhancement on T1WI-A, representing lesion with peripheral enhancement, (h) Enhancement pattern (gradual and filling, persistent enhancement, and wash-in and -out pattern); (i) Visible vessel penetration within the lesion, including branches of the hepatic arteries, as well as the portal and hepatic veins; (j) Peripheral hepatic enhancement, described as hepatic parenchyma enhancement around the lesion at any phase; (k) The liver imaging reporting and data system (LI-RADS), dependent on LI-RADSv2018 [28]. All MR images were independently analyzed by three MVI status-blinded abdominal radiologists (X.L.Q., X.J.M., and C.Y. with 6, 11, and 16 years of experience, respectively). Qualitative data were judged and recorded by three radiologists separately, and different results were decided by the most experienced radiologist (C.Y.). Quantitative data were averaged after three independent measurements by X.J.M.

### Radiomics analysis

#### Workflow

**Fig. 2:**
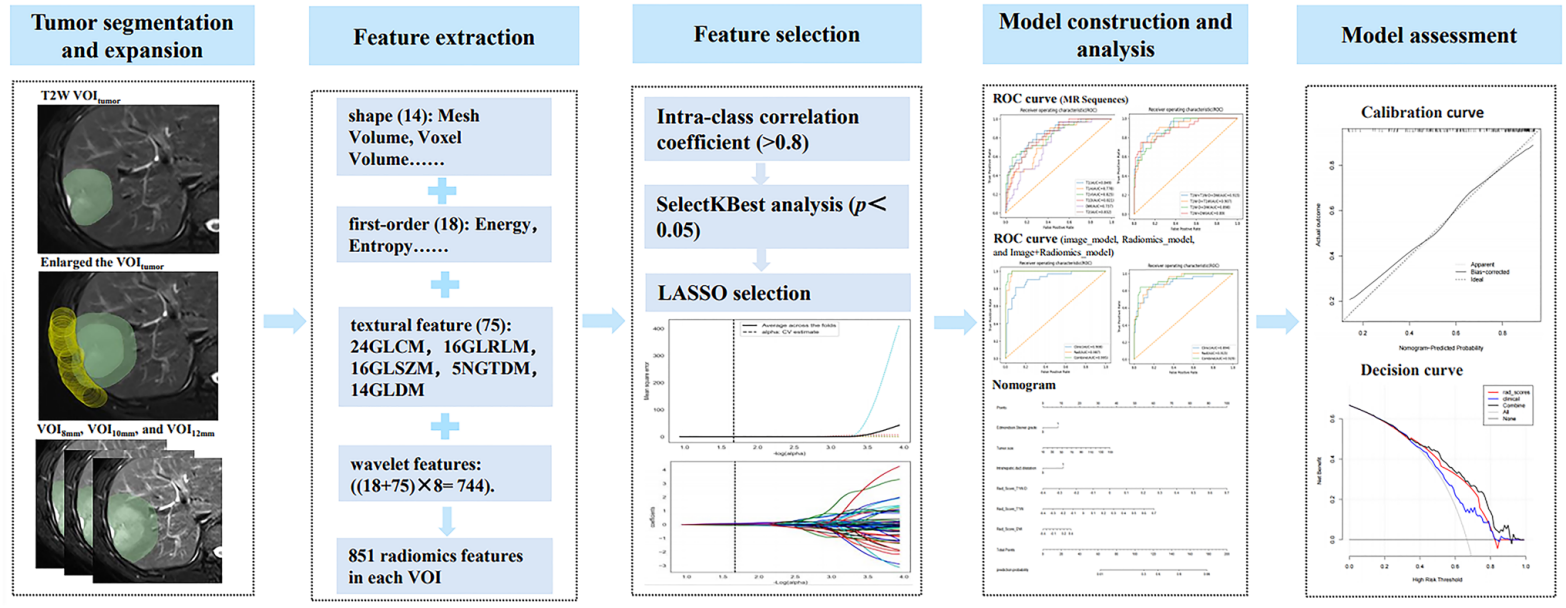
Study flowchart of the radiomics analysis

The workflow of radiomics analysis included tumor segmentation and expansion, feature extraction and selection, model construction and analysis, and model assessment (Fig. 2).

#### Tumor segmentation and VOI expansion

VOIs were drawn on six sequences: DWI, T2WI-FS, T1WI, T1WI-A, T1WI-V, and T1WI-D. First, the VOI_tumor_ was the tumor volume segmentation obtained by manually delineating in the ITK-SNAP software (http://www.itksnap.org/pmwiki/pmwiki.php) [29] by two abdominal radiologists (X.L.Q. and J.Z., both with 6 years of MRI experience), thus avoiding the inclusion of non-neoplastic components such as peripheral hepatic enhancement. The VOI_tumor_ on T1WI was manually delineated, while the VOI_tumor_ on T1WI-A, T1WI-V, or T1WI-D was obtained by replicating VOI on T1WI after registering their images with the T1WI sequence respectively because their field of view (FOV) and image layers were matched. The same method was used in the images on T2WI and DWI. If FOVs or image layers mismatch, VOIs of other sequences were manually delineated according to the VOI_tumor_ on T1WI. Then, the peritumoral region of 8, 10, and 12 mm was automatically expanded by using the “Margin” module of the 3D-Slicer 4.11.20210226 software (https://www.slicer.org/), and the volume outside the hepatic contour was manually removed by two radiologists (Q.W. and R.L.Z. with 5 and 8 years of MRI experience, respectively). The obtained expansion VOIs, which included tumor and peritumoral region, were denoted as VOI_8mm_, VOI_10mm_, and VOI_12mm_, respectively. If the two radiologists had input on the VOI definition, the third radiologist (X.J.M.) was invited to resolve differences together, and differences were resolved by majority, consensus, or averaging. The process of the VOI delineation, expansion, and replication is shown in Supplemental Fig. S1.

#### Feature extraction and selection

Radiomics features were extracted from VOIs by using “Radiomics” of 3D-Slicer software (https://www.radiomics.io/pyradiomics.html). This package automatically developed a pool of radiomics features from each VOI.

To evaluate the repeatability of VOI delineation and enlargement, 30 cases were randomly selected from the collected data, and the images were delineated by reader 1 (X.L.Q.) and reader 2 (X.J.M.), respectively. The interclass correlation coefficient was calculated to assess the inter-observer agreement. Reader 1 delineated the VOI of the same 30 cases after a month, and the intraclass correlation coefficient was calculated to assess the intra-observer agreement. Radiomics features with a threshold greater than 0.8 were retained and used for dimensionality reduction analysis.

The dimensionality reduction process was divided into two steps (Supplemental Table S2): First, the selected features with *p*-value < 0.05 in the SelectKBest analysis were selected; next, the least absolute shrinkage and selection operator (LASSO) algorithm was employed to obtain the final radiomics features and feature coefficients. The lambda value (*λ*) determines which features make the model optimal, and tenfold cross-validation is used to find the best *λ*: the *λ* corresponding to the minimum mean square error (MSE) determines the useful features to be included in the model. Finally, Rad-score was calculated according to the following formula.

$${\text{Rad}}\;{\text{score}} = {\text{Intercept}} + \sum\limits_{i = 1}^{n} {{\text{coefficients}}\;[i] \times {\text{Feature}}\;[i]}$$

### Model construction and analysis

The image model was constructed by independent imaging features selected via uni- and multivariate logistic regression (LR) analysis. 24 single-sequence models of 6 MRI sequences based on 4 VOI-subgroups (including VOI_tumor_, VOI_8mm_, VOI_10mm,_ and VOI_12mm_) were constructed by corresponding optimal features. Meanwhile, based on the optimal VOI-subgroup, various combinations of the top three optimal sequences were constructed and compared to select the best combination for constructing the final radiomics model. Finally, independent imaging characteristics and Rad-scores of the three optimal sequences were employed for nomogram construction. All models were constructed using LR and support vector machine (SVM) classifiers.

### Model assessment

The predictive efficacy was evaluated by receiver operating characteristics curve (ROC) analysis. The AUC, sensitivity, and specificity were calculated. The Delong test was used to compare the predictive efficacy of different models. The Hosmer–Lemeshow test was used to compare the agreement between nomogram-estimated MVI status and the real MVI status. The calibration curves were constructed to evaluate the goodness-of-fit of the nomogram. The decision curve analysis (DCA) assessed the net benefits of models at a range of risk thresholds.

### Statistical analysis

The baseline between TC and VC was evaluated using the Student’s *t*-test, Mann–Whitney *U* test, Wilcoxon test, Chi-square, or Fisher’s exact test, as appropriate. Univariate and multivariate analysis identified independent clinical and imaging MVI predictors. SPSS version 25.0 (SPSS Inc) was employed for all data analyses. The Python version 3.6 Sklearn package was used to dimensionally reduce the radiomics features, prior to plotting the ROC curve. The AUC, accuracy, sensitivity, and specificity were assessed. A two-tailed *p*-value < 0.05 was set as the significance threshold. Lastly, several plots were generated using the R software (version 4.1.2).

## Results

### Demographic and clinicoradiologic profiles of patients

The inter- and intra-class correlation coefficients of the tumor imaging profiles were both significant (> 0.8). Table 1 summarizes the comparisons of the MVI status, demographic, clinical, and imaging characteristics in TC and VC. 160 patients (117 males and 43 females, 60.29 ± 11.43 years old, age range: 29–86 years old) were enrolled in this study. There were 79 MVI- and 32 MVI+ patients in the TC (*n* = 111), and 35 MVI- and 14 MVI+ patients in the VC (*n* = 49). No obvious differences existed in the MVI status between TC and VC (*p* = 1.000). Based on multivariate analysis, tumor size (*p* = 0.01; OR = 1.03, 95% CI 1.01–1.05) and intrahepatic ductal dilatation (*p* = 0.02; OR = 3.04, 95% CI 1.21–7.63) were independent indicators of MVI (Table 2). The Image model was constructed with these two independent predictors. Examples of the representative image characteristics are shown in Supplemental Fig. S2.

**Table 2:** Univariate and multivariate analysis of predictive characteristics related with MVI status

Characteristics	Univariate			Multivariate		
	OR	95% CI	*p*-value	OR	95% CI	*p*-value
Age	1.00	0.97–1.04	0.95			
Gender	0.93	0.37–2.32	0.87			
HBV	1.14	0.50–2.62	0.75			
AFP	0.80	0.20–3.19	0.76			
CEA	1.94	0.76–4.94	0.17			
CA199	1.89	0.82–4.33	0.13			
Edmondson-Steiner grade	3.13	1.09–9.02	**0.03**	2.25	0.73–6.90	0.16
Tumor size	1.03	1.01–1.05	**< 0.001**	1.03	1.01–1.05	**0.01**
Tumor morphology	1.51	0.87–2.61	0.15			
SI on T1WI	4.32	0.53–34.91	0.17			
SI on T2WI-FS	1.22	0.12–12.22	0.86			
Target sign on T2WI-FS	1.45	0.60–3.50	0.41			
Target sign on DWI	1.72	0.75–3.96	0.20			
Rim enhancement on T1WI-A	1.05	0.39–2.83	0.92			
Enhancement pattern	0.86	0.38–1.96	0.72			
LI-RADS	0.77	0.38–1.55	0.46			
Intrahepatic duct dilatation	4.06	1.71–9.64	**< 0.001**	3.04	1.21–7.63	**0.02**
Hepatic capsular retraction	1.44	0.63–3.30	0.39			
Visible vessel penetration	1.29	0.56–2.96	0.55			
Peripherally hepatic enhancement	0.60	0.26–1.37	0.23			

The bold values are statistically significant with *p* < 0.05

#### Radiomics features

The radiomics features extracted were stratified into the First Order, Shape, Gray-Level Co-occurrence Matrix (GLCM), Gray-Level Size Zone Matrix (GLSZM), Gray-Level Run Length Matrix (GLRLM), Neighboring Gray Tone Difference Matrix (NGTDM), and Gray-Level Dependence Matrix (GLDM) features. The radiomics features on each three-dimensional segmentation were grouped as follows: (1) shape (*n* = 14), first-order (*n* = 18), textural profile (*n* = 75), and wavelet profile (*n* = 744). Ultimately, 851 features were obtained from each VOI per sequence per patient. The numbers of the selected features of each VOI of every single sequence during the procedure of feature selection are provided in Supplemental Table S2.

### Performance of radiomics features from single MRI sequences

Table 3 summarizes the AUCs of four VOI-subgroups in six single-sequence models, and matched ROCs are provided in Supplemental Fig. S3. In the same sequence, the best predictive efficiencies are obtained from the VOI_10mm_ subgroup. Next, three radiomics models based on VOI_10mm_ of T1WI-D, T1WI, and DWI sequences exhibited ideal and stable predictive efficacy with AUCs > 0.8. Delong test between SVM and LR of single-sequences based on multiple VOIs is provided in Supplemental Table S3. In terms of different VOIs and sequences, all of the LR results were better than SVM in the TC and VC, and most of them were statistically significant. To sum up, the top three optimal sequences (T1WI-D, T1WI, DWI) based on the VOI_10mm_ by LR were finally selected for further analysis. The selection procedure of robust features of each VOI_10mm_ of the three optimal sequences is provided in Supplemental Fig. S4. And the detail of robust features of each VOI_10mm_ of the three optimal sequences is listed in Supplemental Table S4. Lastly, the performance of every single sequence based on VOI_10mm_ in predicting MVI status by using LR is provided in Supplemental Table S5.

**Table 3:** The performance of single-sequence models based on multiple VOIs and multi-sequence combination models based on VOI_10mm_ in predicting MVI status

Sequences	Classifiers and Cohorts	AUCs			
		VOI_tumor_	VOI_8mm_	VOI_10mm_	VOI_12mm_
T2WI	SVM(TC/VC)	0.715/0.614	0.728/0.632	0.773/0.676	0.665/0.541
	LR(TC/VC)	0.753/0.616	0.773/0.639	0.799/0.697	0.732/0.611
T1WI-V	SVM(TC/VC)	0.725/0.649	0.743/0.663	0.773/0.690	0.716/0.606
	LR(TC/VC)	0.743/0.667	0.767/0.701	0.788/0.739	0.721/0.663
T1WI-D	SVM(TC/VC)	0.766/0.680	0.792/0.694	0.798/0.767	0.778/0.678
	LR(TC/VC)	0.849/0.780	0.811/0.784	0.850/**0.831**	0.838/0.745
T1WI-A	SVM(TC/VC)	0.726/0.632	0.744/0.639	0.779/0.690	0.705/0.611
	LR(TC/VC)	0.729/0.645	0.773/0.691	0.784/0.722	0.714/0.618
T1WI	SVM(TC/VC)	0.737/0.657	0.786/0.659	0.771/0.745	0.719/0.659
	LR(TC/VC)	0.809/0.722	0.811/0.729	0.823/**0.814**	0.807/0.718
DWI	SVM(TC/VC)	0.733/0.620	0.765/0.629	0.790/0.706	0.763/0.621
	LR(TC/VC)	0.788/0.702	0.801/0.704	0.808/**0.808**	0.781/0.690
T1WI+DWI	SVM(TC/VC)	/	/	0.890/0.808	/
	LR(TC/VC)	/	/	0.963/0.831	/
T1WI-D+DWI	SVM(TC/VC)	/	/	0.898/0.812	/
	LR(TC/VC)	/	/	0.968/0.843	/
T1WI-D+T1WI	SVM(TC/VC)	/	/	0.907/0.814	/
	LR(TC/VC)	/	/	0.974/0.853	/
T1WI+T1WI-D+DWI	SVM(TC/VC)	/	/	0.915/0.820	/
	LR(TC/VC)	/	/	**0.987/0.859**	/

The bold values exhibit ideal and stable predictive efficacy with AUCs > 0.8 of the top three optimal sequences and the best combination sequence based on the optimal VOI-subgroup

*TC* training cohort, *VC* validation cohort

### Performance of radiomics features from multiple MRI sequences

**Fig. 3:**
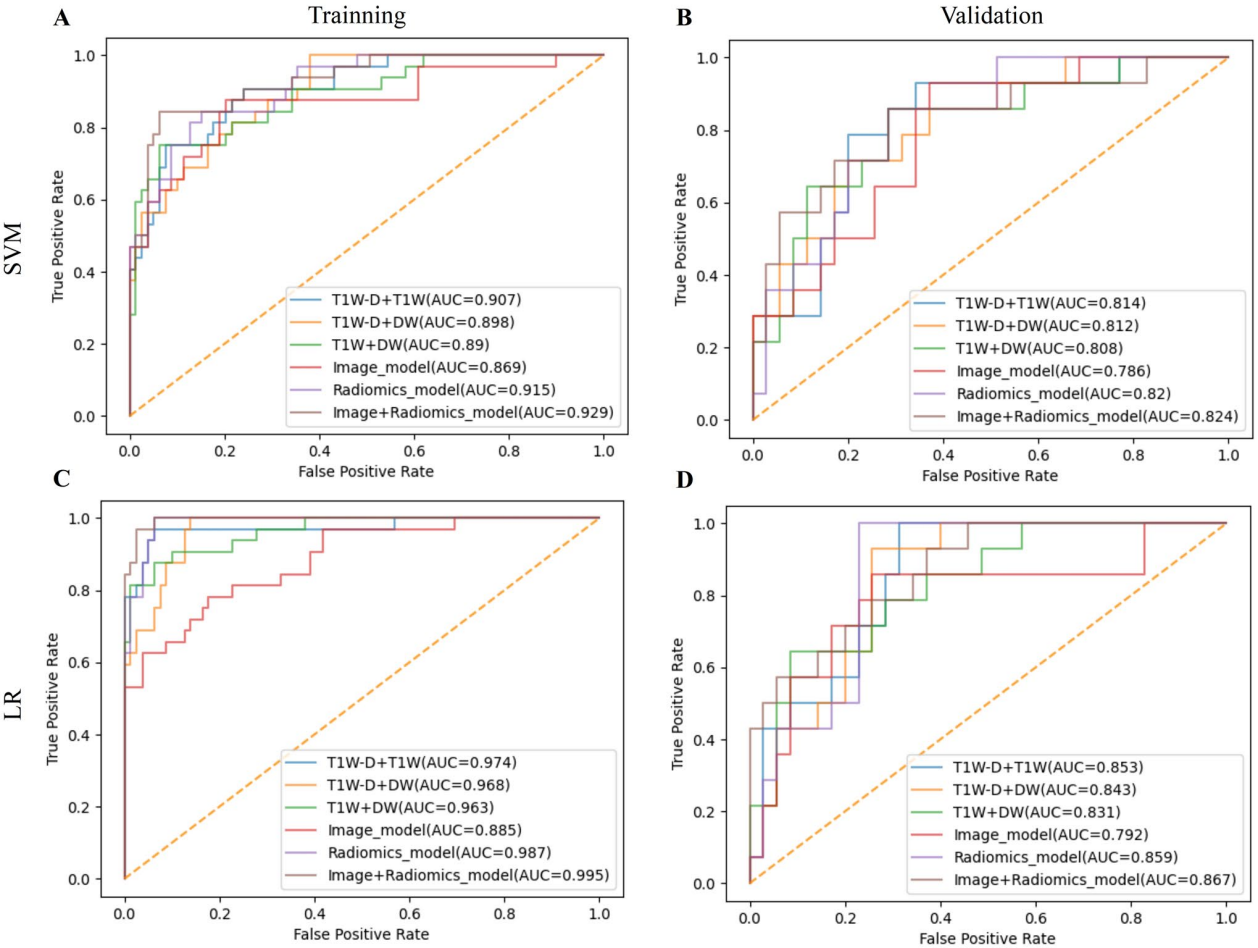
ROCs of various models that predict MVI status in training cohort (TC) and validation cohort (VC) by support vector machine (SVM) and logistic regression (LR) classifiers

In the VOI_10mm_ subgroup, one three-sequence combination model and three pairwise combination models based on the DWI, T1WI, and T1WI-D images were constructed. The three-sequence combination model performed better than the three pairwise combination models in predicting MVI status in both TC and VC (AUC_TC_ = 0.987, AUC_VC_ = 0.859) (Tables 3, 4, and Fig. 3). Therefore, the three-sequence combination model based on VOI_10mm_ was regarded as the Radiomics model.

**Table 4:** The comparison of various models in training and validation cohorts

Models for comparison	Classifier	*P*_Training cohort_	*P*_Validation cohort_
T1WI+DWI VS T1WI-D+DWI	SVM	0.546	0.425
	LR	0.453	0.426
T1WI-D+T1WI VS T1WI-D+DWI	SVM	0.517	0.336
	LR	0.213	0.465
T1WI-D+DWI VS T1WI-D+T1WI	SVM	0.417	0.313
	LR	0.362	0.497
Radiomics model VS T1WI+DWI	SVM	0.083	0.252
	LR	**0.046**	0.278
Radiomics model VS T1WI-D+DWI	SVM	0.136	0.294
	LR	0.057	0.351
Radiomics model VS T1WI-D+T1WI	SVM	**0.023**	0.318
	LR	**0.044**	**0.033**
Radiomics model VS Image model	SVM	**< 0.001**	**0.015**
	LR	**< 0.001**	**0.023**
Image+Radiomics model VS Image model	SVM	**0.019**	**0.032**
	LR	**0.024**	**0.040**
Image+Radiomics model VS Radiomics model	SVM	**0.043**	**0.011**
	LR	**0.037**	0.071

All sequences in the table are based on VOI_10mm_ subgroup

The bold values are statistically significant with *p* < 0.05

### Performance and comparison of the combined MVI prediction model

The combined Image+adiomics model achieved excellent predictive efficacy in the TC (AUC = 0.995, 95% CI 0.987–1.000, sensitivity = 0.844, and specificity = 0.987) and VC (AUC = 0.867, 95% CI 0.798–0.921, sensitivity = 0.643, and specificity = 0.800) (Table 5, Fig. 3).

**Table 5:** The performance of MVI prediction models in training cohort and validation cohort

Models	Classifiers	Training cohort (*n* = 111)			Validation cohort (*n* = 49)		
		Sen	Spe	AUC (95% CI)	Sen	Spe	AUC (95% CI)
Image model	SVM	0.469	1.000	0.869 (0.797–0.932)	0.643	0.743	0.786 (0.591–0.843)
	LR	0.781	0.772	**0.885** (0.836–0.947)	0.857	0.686	**0.792** (0.709–0.882)
Radiomics model	SVM	0.812	0.848	0.915 (0.883–0.965)	0.714	0.800	0.820 (0.670–0.889)
	LR	0.625	1.000	**0.987** (0.973–0.997)	1.00	0.743	**0.859** (0.809–0.929)
Image+Radiomics model	SVM	0.844	0.861	0.929 (0.880–0.968)	0.857	0.657	0.824 (0.631–0.853)
	LR	0.844	0.987	**0.995** (0.987–1.000)	0.643	0.800	**0.867** (0.798–0.921)

The bold values are the superior AUC between every model constructed by LR and SVM

The Radiomics model and Image+Radiomics model were both superior to the Image model in the TC (AUCs: 0.987 and 0.995 vs. 0.885, *p* < 0.05) and VC (AUCs: 0.859 and 0.867 vs. 0.792, *p* < 0.05). The Image+Radiomics model was also superior to the Radiomics model in the TC (*p* = 0.037), while there was no statistically significant in the VC (*p* = 0.071). (Tables 4 and 5).

### Development and verification of the nomogram

**Fig. 4:**
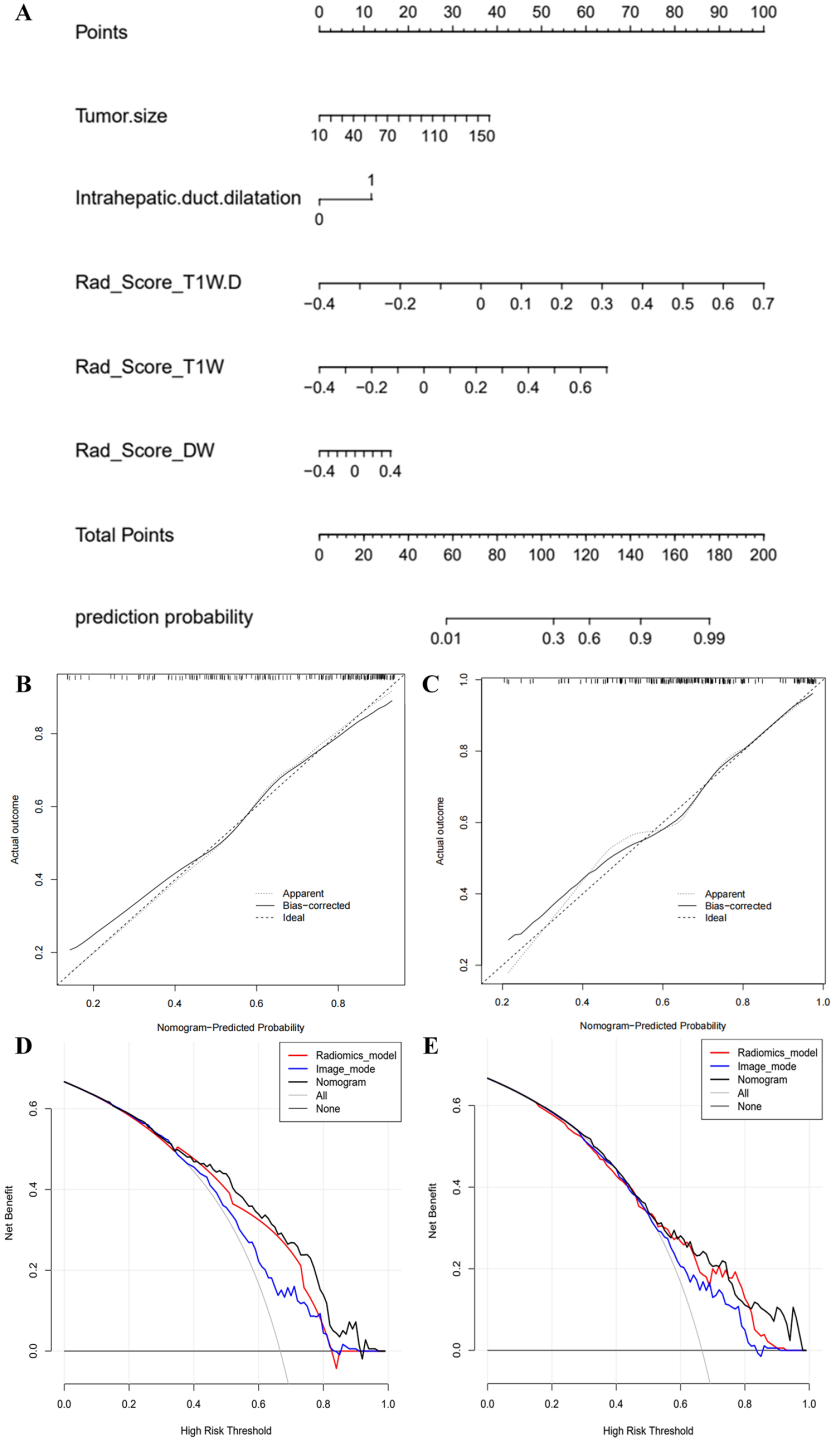
Nomogram of the microvascular invasion (MVI) prediction model, nomogram calibration curves in the training (TC) and validation cohorts (VC), and decision curve analysis (DCA). **A** A nomogram combines two independent imaging predictors (tumor size and intrahepatic duct dilatation), and radiomics features based on VOI_10mm_ of DWI, T1WI, and T1WI-D sequences. **B**, **C** Nomogram calibration curves in the TC and VC. The x-axis represents a nomogram-estimated MVI risk, the y-axis represents the actual MVI risk, and the diagonal dashed line indicates the ideal prediction by an ideal model. **D**, **E** Nomogram DCA curves in the TC and VC. The gray curve is the total benefit of assuming that all intrahepatic cholangiocarcinoma (ICC) patients with MVI; the gray line is the total benefit of assuming no ICC patients with MVI; and the black line is the expected total benefit per patient, according to the nomogram

To visualize the Image+Radiomics model, we developed a nomogram as an assessment tool (Fig. 4A). The formula is as follows:

$$ \begin{aligned} Y-{\text{score}} = & - 0.7950  + 0.0198 *{\text{Image}}\_{\text{Tumor size}}  +0.9062*{\text{Image}}\_{\text{Intrahepatic duct dilatation}} \\ & +7.0557 * {\text{Rad}}\_{\text{score}}\_{\text{T1W-D}}  +4.5624 * {\text{Rad}}\_{\text{score}}\_{\text{T1W}} +1.5527 * {\text{Rad}}\_{\text{score}}\_{\text{DW}}\end{aligned} $$

The nomogram calibration curves demonstrated the goodness-of-fit between the predicted MVI status and the actual MVI status in the TC and VC (Fig. 4B, C). The nomogram decision curves exhibited that the Image model, Radiomics model, and nomogram could obtain net benefits with a risk threshold over 0.3 in the TC and 0.5 in the VC (Fig. 4D, E), and the nomogram showed the highest net benefit.

## Discussion

Our study sought to establish a radiomics nomogram based on the optimal VOI of multi-sequence MRI for preoperative prediction of MVI in ICC tumors. Firstly, multivariate analysis showed that tumor size and intrahepatic ductal dilatation were independent indicators, and the Image model incorporated the two independent indicators. Secondly, we found that the best scale VOI was VOI_10mm_, which included the entire volume of the tumor plus the peritumoral area within a region of 10 mm from the tumor margin, and the best three radiomics models of T1WI-D, T1WI, and DWI sequences based on VOI_10mm_ exhibited ideal and stable predictive efficacy with AUCs > 0.8 by using LR classifier. Therefore, the three-sequence combination model based on VOI_10mm_ is regarded as the final radiomics model. Finally, the final combined Image+Radiomics model achieved excellent predictive efficiency with AUC = 0.995 in the TC and AUC = 0.867 in the VC, and it performed better than the Image model and Radiomics model. To visualize the excellent Image+Radiomics model, the nomogram was developed as an assessment tool, and its clinical significance was further confirmed by the decision curve.

Some studies have reported that MRI-based radiomics models could be useful for predicting MVI in ICC, but the delineated VOIs were based on the lesion itself [19, 20], while in fact, MVI occurs in the peritumoral region of the lesion. Innovatively, we extracted radiomics features from the ICC tumor and multiple peritumoral regions. The best three sequences we selected were consistent with Qian et al. [20] importantly, the nomogram in our study performed better in both TC and VC (AUCs: 0.995 vs. 0.953 in TC, 0.867 vs. 0.861 in VC), which indicated that radiomics features of peritumoral regions may improve the predictive efficacy. The procedure of feature selection in our study (Table S2) also indicates that most of the independent radiomics features occurred in the 10 mm peritumoral region, which fits the pathological definition of MVI. That is to say, our study further confirmed that the MRI radiomics features of the tumoral and the 10 mm peritumoral region can reflect the microenvironment of the ICC tumor to some extent. Based on the principle of MR imaging, the reasons for the optimal three sequences (T1WI-D, T1WI, and DWI) may be as follows: (1) DWI can effectively reflect the molecular movement of tissues and provide valuable imaging information regarding the diffusion and infiltration of cancer cells. (2) T1WI-D, obtained through contrast agent injection, can reflect the perfusion of blood flow and provide certain advantages in evaluating tumor invasion. (3) T1WI can effectively display anatomical structures and tumor morphology, providing various important dimensional and morphological parameters, which are very useful in assessing subtle tumor invasions. Therefore, combining these three sequences can maximize the diagnostic accuracy and precision of MVI in ICC.

Radiomics is well regarded as an essential imaging strategy in oncology [30–33], however, there are still various technical challenges. MVI typically presents in the tumor edge, making the peritumoral tissue the first susceptible tissue, and its vessels become the primary hematogenous path for portal vein tumor thrombosis and metastasis [34, 35], so the radiomics features of the peritumoral tissue must be effectively evaluated. To predict MVI or treatment response, numerous studies examined liver tissue from various areas around the HCC lesions [21–23]. In Chong et al. [21] study involving MRI-based multi-scale radiomics to predict the MVI status of HCC, the final Radiomics model was derived from the optimal multi-sequence fusion in the VOI_tumor+10mm+liver_ subgroup. Chen et al. [23] compared the tumoral and peritumoral radiomics features in different ROI ranges and revealed that the radiomics features extracted from tumoral and 10 mm peritumoral regions, obtained the best predictive efficacy in predicting the response of HCCs to the first trans-arterial chemoembolization treatment. In our study, the multi-VOI models that explored the peritumoral region revealed that the VOI_10mm_ subgroup outperformed the tumor-only and other subgroups, which was similar to the findings in HCC in Chong et al. and Chen et al. studies [21, 23]. It is worth mentioning that the simple radiomics model is usually unstable with abnormally high or low sensitivity or specificity, like our and Zhang’s studies [36]. However, the final combined model includes clinical, image, and radiomics factors and usually performs stable. This is why we extracted independent clinical and image predictors.

Eighteen independent radiomics features were extracted and selected from the best three sequences based on VOI_10mm_ (Supplemental Table S4), some features have been proved to be associated with tumor size (e.g., Maximum 2D Diameter Row), shape (e.g., Flatness), and texture heterogeneity (e.g., Gray-Level Non-Uniformity Normalized), which are essential for predicting MVI. Higher values of the Maximum 2D diameter Row represent larger tumor sizes. This corroborates with prior investigation that reported an association between tumor sizes and MVI risk [24]. Long Run Low Gray-Level Emphasis, Short Run Low Gray-Level Emphasis, and Gray-Level Non-Uniformity Normalized from GLRLM reflect the tumor heterogeneity in gray scales. Tumor heterogeneity may be caused by tumor cellularity, micronecrosis, and inflammation, which further facilitates the MVI process [37, 38]. In addition, wavelet features are strongly associated with survival, which can also quantify intratumoral heterogeneity [39]. In our study, most of the final independent radiomics features were wavelet features, which corroborated with Zhou’s study [19]. Wavelet features may be correlated with tumor morphology, pathophysiology, and proteomics [40].

Previous studies have shown that MVI is one of the factors associated with poor postoperative survival in ICC patients [10], and has been used as one of the reference factors for adjuvant chemotherapy after ICC resection [11]. Our research shows that: (1) MVI can be predicted before surgery with high accuracy, to shunt ICC patients and roughly predict the prognosis; (2) More importantly, if the proportion of independent radiomics features extracted in a certain perifocal region is high, it suggests that there may really exist MVI here, and the region can be extensively resected during surgery, of course, this needs further comparative study with pathology. Totally, preoperative prediction of MVI may help physicians to formulate an overall treatment plan in advance.

### Limitations

Firstly, there was selection bias in this retrospective study. The rate of ICC patients with MVI in this study was 28.75% (46/160), which may be less than the actual positive rate. Secondly, although preoperative imaging for MVI prediction was assessed, the long-term follow-up to observe the association between imaging features, radiomics features, outcome, and survival was needed. Thirdly, to predict MVI status in ICC patients via radiomics, there is currently no unified standard of tumoral and the range of peritumoral tissue VOIs delineation for extracting radiomics features. Fourthly, 8, 10, and 12 mm peritumoral regions were automatically expanded by the “Margin” module, thus the peritumoral regions are basically the same in all sequences. But it is relatively similar actually because the respiratory motion artifact is inevitable. Finally, nine 1.5T and 3.0T MRI scanners from four manufacturers with different parameters were used in our research, which may introduce bias.

### Conclusion

We developed and validated a nomogram combining tumor size, intrahepatic ductal dilatation, and Radiomics models of T1WI, T1WI-D, and DWI sequences based on VOI_10mm_ (including tumoral and 10 mm peritumoral region), which may be an indicator to predict presurgical MVI status in MF-ICC.

### Supplementary Information

Below is the link to the electronic supplementary material.

Supplementary file1 (DOCX 1432 KB)

## References

[1] Squadroni M, Tondulli L, Gatta G, Mosconi S, Beretta G, Labianca R (2017). Cholangiocarcinoma. Crit Rev Oncol Hematol.

[2] Sung H, Ferlay J, Siegel RL (2021). Global cancer statistics 2020: GLOBOCAN estimates of incidence and mortality worldwide for 36 cancers in 185 countries. CA Cancer J Clin.

[3] Utada M, Ohno Y, Tamaki T, Sobue T, Endo G (2014). Long-term trends in incidence and mortality of intrahepatic and extrahepatic bile duct cancer in Japan. J Epidemiol.

[4] Bergquist A, von Seth E (2015). Epidemiology of cholangiocarcinoma. Best Pract Res Clin Gastroenterol.

[5] Lim JH (2003). Cholangiocarcinoma: morphologic classification according to growth pattern and imaging findings. AJR Am J Roentgenol.

[6] Hirohashi K, Uenishi T, Kubo S (2002). Macroscopic types of intrahepatic cholangiocarcinoma: clinicopathologic features and surgical outcomes. Hepatogastroenterology.

[7] Everhart JE, Ruhl CE (2009). Burden of digestive diseases in the United States part III: liver, biliary tract, and pancreas. Gastroenterology.

[8] Wang K, Zhang H, Xia Y, Liu J, Shen F (2017). Surgical options for intrahepatic cholangiocarcinoma. Hepatobiliary Surg Nutr.

[9] Rahnemai-Azar AA, Weisbrod AB, Dillhoff M, Schmidt C, Pawlik TM (2017). Intrahepatic cholangiocarcinoma: current management and emerging therapies. Expert Rev Gastroenterol Hepatol.

[10] Ali SM, Clark CJ, Mounajjed T (2015). Model to predict survival after surgical resection of intrahepatic cholangiocarcinoma: the Mayo clinic experience. HPB (Oxford).

[11] Tsukamoto M, Yamashita YI, Imai K (2017). Predictors of cure of intrahepatic cholangiocarcinoma after hepatic resection. Anticancer Res.

[12] Cong WM, Bu H, Chen J (2016). Practice guidelines for the pathological diagnosis of primary liver cancer: 2015 update. World J Gastroenterol.

[13] Surov A, Pech M, Omari J (2021). Diffusion-weighted imaging reflects tumor grading and microvascular invasion in hepatocellular carcinoma. Liver Cancer.

[14] Shao C, Chen J, Chen J, Shi J, Huang L, Qiu Y (2017). Histological classification of microvascular invasion to predict prognosis in intrahepatic cholangiocarcinoma. Int J Clin Exp Pathol.

[15] Tang Z, Liu WR, Zhou PY (2019). Prognostic value and predication model of microvascular invasion in patients with intrahepatic cholangiocarcinoma. J Cancer.

[16] Zhou Y, Wang X, Xu C (2019). Mass-forming intrahepatic cholangiocarcinoma: can diffusion-weighted imaging predict microvascular invasion?. J Magn Reson Imaging.

[17] Ma X, Liu L, Fang J (2020). MRI features predict microvascular invasion in intrahepatic cholangiocarcinoma. Cancer Imaging.

[18] Lambin P, Leijenaar RTH, Deist TM (2017). Radiomics: the bridge between medical imaging and personalized medicine. Nat Rev Clin Oncol.

[19] Zhou Y, Zhou G, Zhang J, Xu C, Wang X, Xu P (2021). Radiomics signature on dynamic contrast-enhanced MR images: a potential imaging biomarker for prediction of microvascular invasion in mass-forming intrahepatic cholangiocarcinoma. Eur Radiol.

[20] Qian X, Lu X, Ma X (2022). A multi-parametric radiomics nomogram for preoperative prediction of microvascular invasion status in intrahepatic cholangiocarcinoma. Front Oncol.

[21] Chong HH, Yang L, Sheng RF (2021). Multi-scale and multi-parametric radiomics of gadoxetate disodium-enhanced MRI predicts microvascular invasion and outcome in patients with solitary hepatocellular carcinoma ≤ 5 cm. Eur Radiol.

[22] Xu X, Zhang HL, Liu QP (2019). Radiomic analysis of contrast-enhanced CT predicts microvascular invasion and outcome in hepatocellular carcinoma. J Hepatol.

[23] Chen M, Cao J, Hu J (2021). Clinical-radiomic analysis for pretreatment prediction of objective response to first transarterial chemoembolization in hepatocellular carcinoma. Liver Cancer.

[24] Lei Z, Li J, Wu D (2016). Nomogram for preoperative estimation of microvascular invasion risk in hepatitis B virus-related hepatocellular carcinoma within the Milan criteria. JAMA Surg.

[25] Washington MK, Berlin J, Branton PA (2010). Protocol for the examination of specimens from patients with carcinoma of the intrahepatic bile ducts. Arch Pathol Lab Med.

[26] Zhou J, Sun HC, Wang Z (2018). Guidelines for diagnosis and treatment of primary liver cancer in China (2017 edition). Liver Cancer.

[27] Lewis S, Besa C, Wagner M (2018). Prediction of the histopathologic findings of intrahepatic cholangiocarcinoma: qualitative and quantitative assessment of diffusion-weighted imaging. Eur Radiol.

[28] Marrero JA, Kulik LM, Sirlin CB (2018). Diagnosis, staging, and management of hepatocellular carcinoma: 2018 practice guidance by the American association for the study of liver diseases. Hepatology.

[29] Yushkevich PA, Piven J, Hazlett HC (2006). User-guided 3D active contour segmentation of anatomical structures: significantly improved efficiency and reliability. Neuroimage.

[30] Aerts HJ, Velazquez ER, Leijenaar RT (2014). Decoding tumour phenotype by noninvasive imaging using a quantitative radiomics approach. Nat Commun.

[31] Kickingereder P, Götz M, Muschelli J (2016). Large-scale radiomic profiling of recurrent glioblastoma identifies an imaging predictor for stratifying anti-angiogenic treatment response. Clin Cancer Res.

[32] Gillies RJ, Kinahan PE, Hricak H (2016). Radiomics: images are more than pictures, they are data. Radiology.

[33] Huang Y, Liu Z, He L (2016). Radiomics signature: a potential biomarker for the prediction of disease-free survival in early-stage (I or II) non-small cell lung cancer. Radiology.

[34] Hu HT, Shen SL, Wang Z (2018). Peritumoral tissue on preoperative imaging reveals microvascular invasion in hepatocellular carcinoma: a systematic review and meta-analysis. Abdom Radiol (NY).

[35] Hu H, Zheng Q, Huang Y (2017). A non-smooth tumor margin on preoperative imaging assesses microvascular invasion of hepatocellular carcinoma: a systematic review and meta-analysis. Sci Rep.

[36] Zhang S, Huang S, He W (2022). Radiomics-based preoperative prediction of lymph node metastasis in intrahepatic cholangiocarcinoma using contrast-enhanced computed tomography. Ann Surg Oncol.

[37] Yang L, Gu D, Wei J (2019). A radiomics nomogram for preoperative prediction of microvascular invasion in hepatocellular carcinoma. Liver Cancer.

[38] Wang WT, Yang L, Yang ZX (2018). Assessment of microvascular invasion of hepatocellular carcinoma with diffusion kurtosis imaging. Radiology.

[39] Chen S, Zhu Y, Liu Z, Liang C (2017). Texture analysis of baseline multiphasic hepatic computed tomography images for the prognosis of single hepatocellular carcinoma after hepatectomy: a retrospective pilot study. Eur J Radiol.

[40] Liang W, Xu L, Yang P (2018). Novel nomogram for preoperative prediction of early recurrence in intrahepatic cholangiocarcinoma. Front Oncol.

