# Supplementary material for: Radiomics nomogram based on optimal VOI of multi-sequence MRI for predicting microvascular invasion in intrahepatic cholangiocarcinoma
Source: Radiol Med. 2023 Sep 7;128(11):1296–309. doi: 10.1007/s11547-023-01704-8 (PMC10620280; doi:10.1007/s11547-023-01704-8)
Supplement: Supplementary file 1 — Supplementary file1 (DOCX 1432 KB) [file 11547_2023_1704_MOESM1_ESM.docx]

Supplemental Materials

**Supplemental Table S1.** MR imaging sequences and parameters from different scanners.

| Parameters | T2WI-FS | T1WI-FS tra | DWI |
| --- | --- | --- | --- |
| **1.5T UIHMR 560** |  |  |  |
| Repetition time (msec) | 2693 | 4.4 - 4.5 | 2807 |
| Echo time (msec) | 85.58 | 2.1- 2.2 | 75.7 |
| Matrix size | 201×288 | 192×256 | 115×128 |
| Field of view (mm^2^) | 380×360 | 400×280 | 380×300 |
| Slice thickness (mm) | 6 | 3.5 | 6 |
| Slice gap (mm) | 1.2 | 0 | 1.2 |
| Average | 1 | 1 | 4 |
| **3.0-T UIHMR770** |  |  |  |
| Repetition time (msec) | 2000 | 3.3 | 4165 |
| Echo time (msec) | 106.2 | 1.5 | 66.3 |
| Matrix size | 256×256 | 320×216 | 128×101 |
| Field of view (mm^2^) | 346×346 | 270×270 | 300×300 |
| Slice thickness (mm) | 6-7 | 3 | 6-7 |
| Slice gap (mm) | 1.8-2.1 | 0 | 1.8-2.1 |
| Average | 1 | 1 | 5 |
| **Avanto 1.5-T Siemens** |  |  |  |
| Repetition time (msec) | 3000 - 3500 | 5.04 | 2400 - 2600 |
| Echo time (msec) | 70 - 84 | 2.31 | 66 |
| Matrix size | 256×173 | 256×125 | 128×112 |
| Field of view (mm^2^) | 285×214 - 308×380 | 285×214 - 308×380 | 285×214 - 308×380 |
| Slice thickness (mm) | 5 - 7 | 3 - 4 | 5 - 7 |
| Slice gap (mm) | 1 - 2.1 | 0 | 1 - 2.1 |
| Average | 1 | 1 | 1 |
| **Aera** **1.5-T Siemens** |  |  |  |
| Repetition time (msec) | 3500 | 4.38 | 3200 |
| Echo time (msec) | 84 | 1.93 | 56 |
| Matrix size | 320×224 | 320×240 | 128×128 |
| Field of view (mm^2^) | 380×308 | 380×297 | 380×308 |
| Slice thickness (mm) | 5.5 | 3 - 4 | 5.5 |
| Slice gap (mm) | 1.1 | 0 | 1.1 |
| Average | 1 | 1 | 1 |
| **Verio 3.0-T Siemens** |  |  |  |
| Repetition time (msec) | 2000 -3 000 | 4.17 | 3400 |
| Echo time (msec) | 83 | 1.43 | 70 |
| Matrix size | 320×165 | 352×200 | 128×80 |
| Field of view (mm^2^) | 285×380 - 330×380 | 285×380 - 330 ×380 | 285×380 - 330 ×380 |
| Slice thickness (mm) | 5.5 | 3 | 6 |
| Slice gap (mm) | 1.1 | 0 | 1.8 |
| Average | 1 | 1 | 4 |
| **3.0-T GE MR750** |  |  |  |
| Repetition time (msec) | 4500 | 3.54 | 5454 |
| Echo time (msec) | 88 | 1.67 | 49 |
| Matrix size | 320×224 - 448×448 | 212×170 - 256×192 | 130×96 |
| Field of view (mm^2^) | 380×380 - 400×400 | 380×380 - 400×400 | 380×380 - 400×400 |
| Slice thickness (mm) | 6 - 6.5 | 4.8-5 | 6 - 8 |
| Slice gap (mm) | 1-2 | 0 | 1-2 |
| Average | 2 | 1.33 | 1 |
| **Philips Achieva 1.5-T** |  |  |  |
| Repetition time (msec) | 1500-3000 | 4.2 | 1000-2500 |
| Echo time (msec) | 80-100 | 2.0 | 60-80 |
| Voxel | 1.2×1.2 - 1.4×1.8 | 3×3 | 2×2 |
| Field of view (mm^2^) | 350×350 - 400×400 | 350×350 - 400×400 | 350×350 - 400×400 |
| Slice thickness (mm) | 5 - 7 | 4 | 5 - 7 |
| Slice gap (mm) | 1 | -2 | 1 |
| Average | 1 | 1 | 6 |

**Supplemental Table S2.** The numbers of the selected features of each VOI of every single sequence during the procedure of feature selection.

| Sequences | Input features | Feature numbers (N) | | |
| --- | --- | --- | --- | --- |
|  |  | Intra-class correlation  Coefficient (>0.8) | SelectKBest (*p*<0.05) | LASSO selection |
|  |  | VOI_tumor_/VOI_8mm_/VOI_10mm_/VOI_12mm_ | VOI_tumor_/VOI_8mm_/VOI_10mm_/VOI_12mm_ | VOI_tumor_/VOI_8mm_/VOI_10mm_/VOI_12mm_ |
| T2WI | 851 | 791/802/843/785 | 59/12/13/13 | 12/2/2/7 |
| T1WI-V | 851 | 818/832/793/817 | 128/27/34/33 | 12/5/8/6 |
| T1WI-D | 851 | 781/842/823/835 | 183/19/97/56 | 13/8/**6**/13 |
| T1WI-A | 851 | 758/789/743/801 | 77/29/44/23 | 8/8/9/10 |
| T1WI | 851 | 761/752/746/764 | 117/56/60/48 | 14/13/**6**/11 |
| DWI | 851 | 838/802/743/775 | 31/29/28/24 | 9/6/**6**/8 |

VOI, volumetric interest; VOI_tumor_, the entire volume of tumor; VOI_8mm_ or VOI_10mm_ or VOI_12mm_, including the entire volume of the tumor plus the peritumoral region within the distance of 8mm or 10mm or 12mm from the tumor margin, respectively.

**Supplemental Table S3.** Delong test between SVM and LR classifiers of single sequences based on multiple VOI-subgroups.

| Sequences and cohorts | VOI_tumor_ | VOI_8mm_ | VOI_10mm_ | VOI_12mm_ |
| --- | --- | --- | --- | --- |
| T2WI (TC/VC) | **0.023**/0.122 | **0.033**/0.076 | **0.031**/**0.018** | **0.001**/**0.001** |
| T1WI-V (TC/VC) | 0.078/0.067 | **0.002**/**0.043** | 0.238/**0.014** | 0.290/**0.048** |
| T1WI-D (TC/VC) | **<0.001**/**0.01** | **0.040**/**<0.001** | **<0.001**/**<0.001** | 0.031/**<0.001** |
| T1WI-A (TC/VC) | 0.279/0.192 | **0.012**/**0.039** | 0.143/0.243 | 0.118/0.141 |
| T1WI(TC/VC) | **0.046**/**0.003** | **0.010**/**0.004** | **0.043**/**0.045** | **0.019**/**0.025** |
| DWI (TC/VC) | **0.016**/**0.037** | **0.001**/**0.019** | **0.036**/**0.004** | 0.051/**0.018** |

The values listed in the table are p values between support vector machine (SVM) and logistic regression (LR) classifiers. The bold values are statistically significant with p <0.05.

**Supplemental Table S4.** The detailed information of radiomics features based onVOI_10mm_ in T1WI-D, T1WI and DWI images.

| Sequences | Classes | Filters | Features | Coefficients |
| --- | --- | --- | --- | --- |
| T1WI-D VOI_10mm_ | shape | original | Maximum2DDiameterRow | 0.088 |
| T1WI-D VOI_10mm_ | shape | original | Flatness | -0.067 |
| T1WI-D VOI_10mm_ | glszm | wavelet-HLL | GrayLevelNonUniformityNormalized | -0.004 |
| T1WI-D VOI_10mm_ | glrlm | wavelet-LHH | LongRunLowGrayLevelEmphasis | -0.032 |
| T1WI-D VOI_10mm_ | ngtdm | wavelet-LHL | Strength | 0.049 |
| T1WI-D VOI_10mm_ | ngtdm | wavelet-HLL | Strength | 0.035 |
| T1WI VOI_10mm_ | glszm | wavelet-HLH | SmallAreaLowGrayLevelEmphasis | 0.122 |
| T1WI VOI_10mm_ | glszm | wavelet-LLH | ZoneVariance | 0.038 |
| T1W VOI_10mm_ | shape | original | Flatness | -0.056 |
| T1WI VOI_10mm_ | glcm | wavelet-HLL | MaximumProbability | -0.102 |
| T1WI VOI_10mm_ | first order | wavelet-LLL | Kurtosis | 0.056 |
| T1WI VOI_10mm_ | gldm | wavelet-LLL | DependenceEntropy | -0.044 |
| DWI VOI_10mm_ | shape | original | Maximum2DDiameterRow | 0.061 |
| DWI VOI_10mm_ | glcm | wavelet-LHL | Contrast | -0.039 |
| DWI VOI_10mm_ | glcm | Original | InverseVariance | -0.038 |
| DWI VOI_10mm_ | glrlm | wavelet-LHH | ShortRunLowGrayLevelEmphasis | -0.001 |
| DWI VOI_10mm_ | glrlm | wavelet-HHH | GrayLevelNonUniformityNormalized | -0.004 |
| DWI VOI_10mm_ | glszm | wavelet-LLL | SizeZoneNonUniformityNormalized | 0.057 |

Details and formulas of features:

<https://pyradiomics.readthedocs.io/en/latest/features.html#module-radiomics.firstorder>

**Supplemental Table** S**5.** The performance of single-sequence models based on VOI_10mm_ in predicting MVI status by using logistic regression (LR) classifier.

| Sequences |  | Training cohort (*n*=111) | | |  | Validation cohort (*n*=49) | | |
| --- | --- | --- | --- | --- | --- | --- | --- | --- |
|  | Acc | Sen | Spe | AUC (95%CI) | Acc | Sen | Spe | AUC (95%CI) |
| T2WI | 0.613 | 0.531 | 0.646 | 0.657 (0.545-0.756) | 0.714 | 0.714 | 0.714 | 0.753 (0.572-0.821） |
| T1WI-V | 0.712 | 0.656 | 0.734 | 0.773 (0.68-0.844) | 0.612 | 0.786 | 0.543 | 0.739 (0.578-0.798） |
| T1WI-D | 0.802 | 0.312 | 1.000 | **0.850** (0.762-0.923) | 0.735 | 0.214 | 0.943 | **0.831** (0.749-0.894） |
| T1WI-A | 0.685 | 0.594 | 0.722 | 0.726 (0.617-0.805) | 0.694 | 0.857 | 0.629 | 0.722 (0.681-0.861） |
| T1WI | 0.757 | 0.781 | 0.747 | **0.823** (0.738-0.884) | 0.755 | 0.143 | 1.000 | **0.814** (0.721-0.877） |
| DWI | 0.766 | 0.219 | 0.987 | **0.808** (0.708-0.888) | 0.735 | 0.571 | 0.800 | **0.808** (0.715-0.888） |


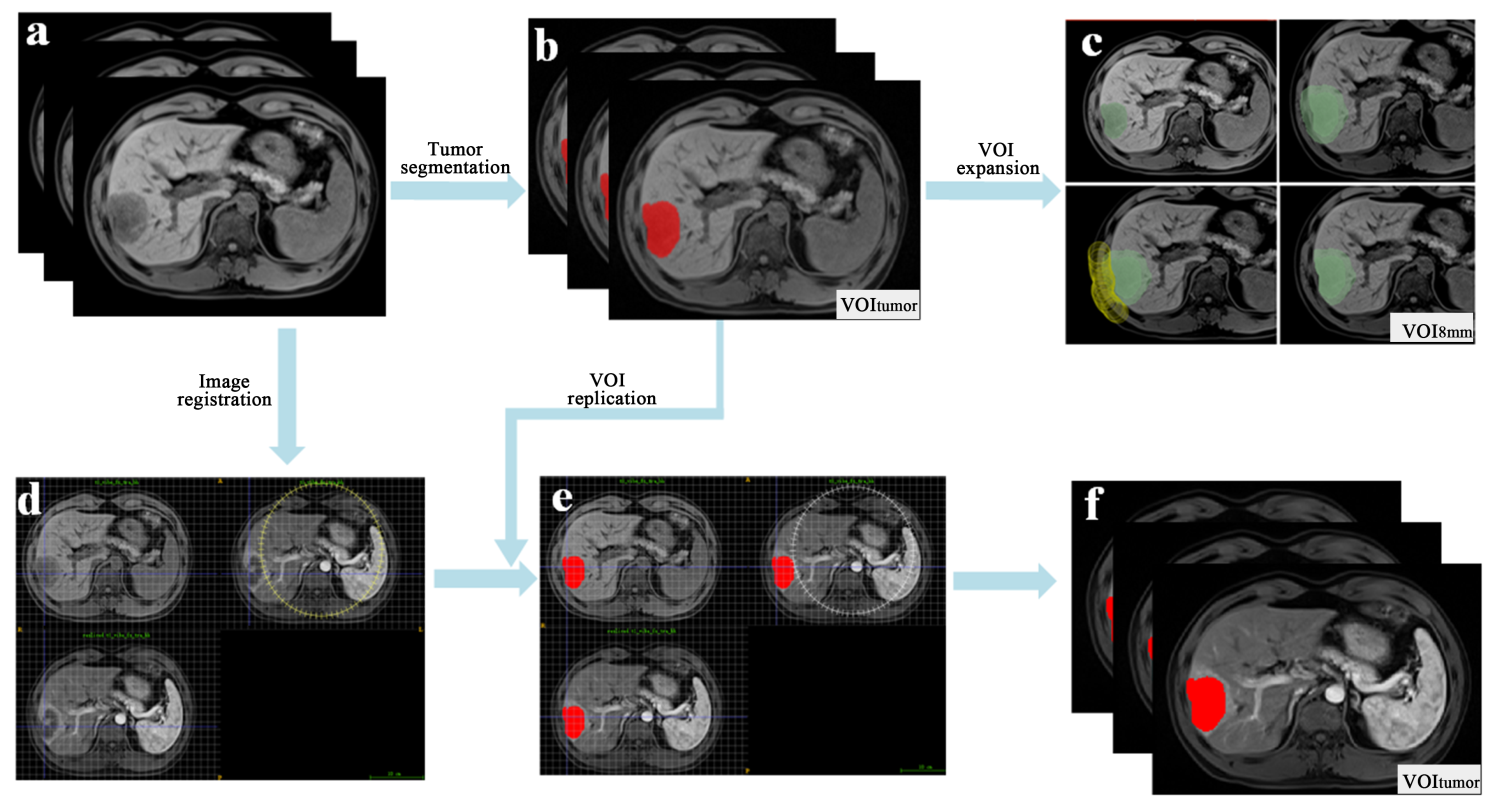


**Supplemental Figure S1.** The process of the VOI delineation, expansion, and replication. a: original image of T1WI. b: VOI_tumor_ manually delineated on T1WI. **c:** VOI_8mm_ was obtained by automatically expanding the 8mm peritumoral range by using the “Margin” module and manually removing the volumes outside the hepatic contour on the 3D-Slicer software. d-f: VOI_tumor_ on T1WI-A was obtained by replicating the VOI_tumor_ on T1WI after registering the images of the two sequences.

**
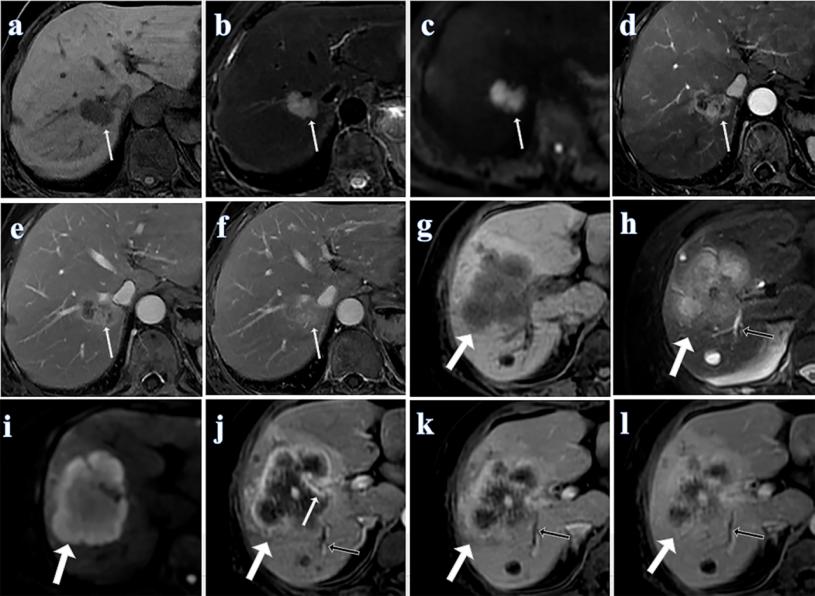
**

**Supplemental Figure S2.** Examples of the representative image characteristics of intrahepatic cholangiocarcinoma (ICC) patients with and without microvascular invasion (MVI). a, g: T1WI, b, h: T2WI, c, i: DWI, d, j: arterial phase (T1WI-A), e, k: portal venous phase (T1WI-V); f, l: delayed phase (T1WI-D). **a-f:** An oval well-defined MVI-negative ICC lesion with a maximum diameter of 34.0 mm in a 68-year-old woman. DWI shows the lesion with hyperintensity. Contrast-enhanced images show rim enhancement on T1WI-A and gradual and filling enhancement on T1WI-V and T1WI-D. **g-l:** A lobulated MVI-positive ICC lesion with a maximum diameter of 77.8 mm in a 66-year-old woman. DWI shows the lesion with the target sign. Contrast-enhanced images show rim enhancement on T1WI-A and gradual and filling enhancement on T1WI-V and T1WI-D, visible branch vessel of the portal vein within the lesion (thin white arrow), and intrahepatic duct dilatation adjacent to tumor (thin black arrow).


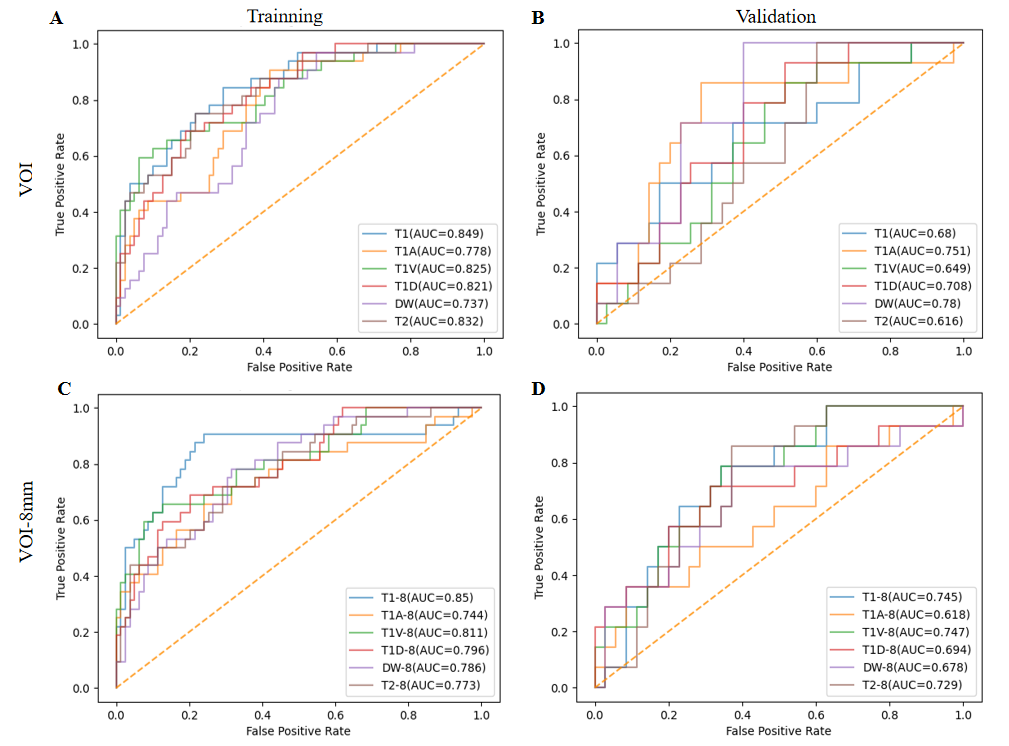


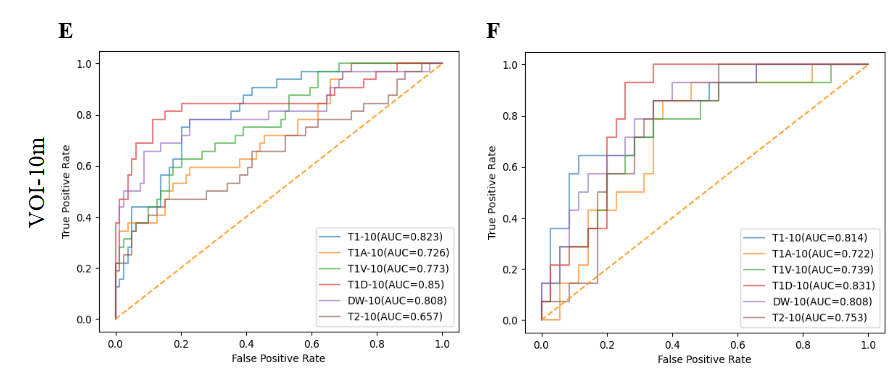


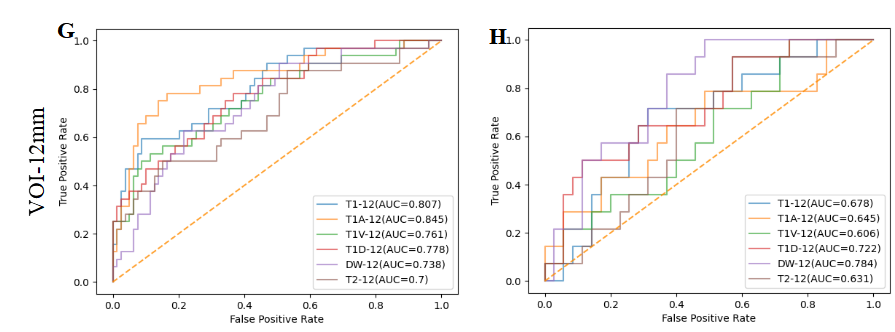


**Supplemental Figure S3.** ROCs of 24 single-sequence models for predicting MVI are plotted by logistic regression (**A, C, E, G**: training cohort, **B, D, F, H**: validation cohort). And 24 single-sequence models of 6 MRI sequences (including T1WI, T1WI-A, T1WI-V, T1WI-D, DWI, T2WI) based on 4 VOI-subgroups (including VOI_tumor_, VOI_8mm_, VOI_10mm_ and VOI_12mm_) are constructed by corresponding optimal features.


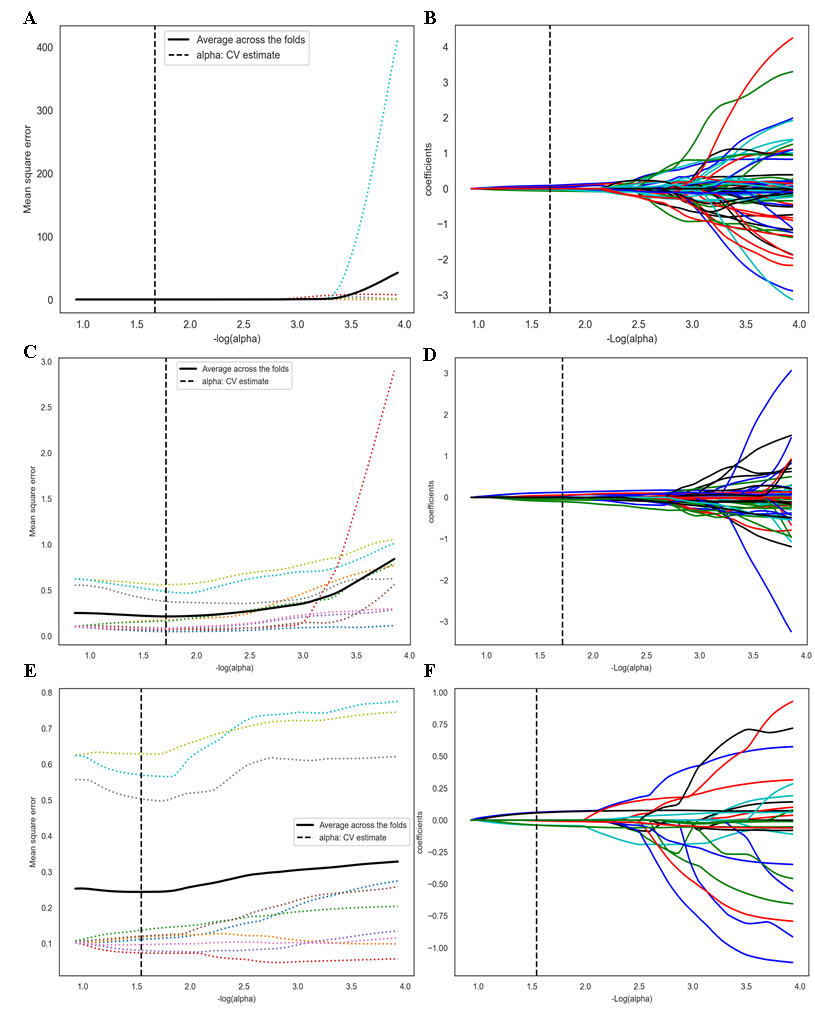


**Supplemental Figure S4.** Radiomics features selection by using MSE (mean square error) path and LASSO regression model on T1-weighted delayed (T1WI-D) phase image (A, B), T1-weighted (T1WI) image (C, D), and diffusion-weighted (DWI) image (E, F) based on VOI_10mm._ The graphs on the left show the mean standard error of the LASSO algorithm during feature screening. 10-fold cross-validation is used to select the best tuning parameter alpha in the LASSO regression model. T1WI-D has an alpha value of 0.022, T1WI has an alpha value of 0.019, and DWI has an alpha value of 0.029. The figures on the right show the non-zero coefficient radiomic features obtained with the optimal tuning parameter alpha, and each colored line represents the change trajectory of each feature coefficient.
